# Resveratrol induces dephosphorylation of Tau by interfering with the MID1-PP2A complex

**DOI:** 10.1038/s41598-017-12974-4

**Published:** 2017-10-23

**Authors:** Susann Schweiger, Frank Matthes, Karen Posey, Eva Kickstein, Stephanie Weber, Moritz M. Hettich, Sandra Pfurtscheller, Dan Ehninger, Rainer Schneider, Sybille Krauß

**Affiliations:** 1 Institute for Human Genetics, University of Mainz, Langenbeckstr. 1, 55131 Mainz, Germany; 2 German Center for Neurodegenerative Diseases (DZNE), Sigmund-Freud-Str.27, 53127 Bonn, Germany; 3 McGovern Medical School at University of Texas in Houston, Department of Pediatrics, 6431 Fannin Street, Houston Texas, 77030 USA; 4 Max-Planck Institute for Molecular Genetics, Department of Human Molecular Genetics, Ihnestr. 73, 14195 Berlin, Germany; 5 Institute of Biochemistry and Center for Molecular Biosciences Innsbruck (CMBI), Innrain 80/82, 6020 Innsbruck, Austria

## Abstract

The formation of paired helical filaments (PHF), which are composed of hyperphosphorylated Tau protein dissociating from microtubules, is one of the pathological hallmarks of Alzheimer’s disease (AD) and other tauopathies. The most important phosphatase that is capable of dephosphorylating Tau at AD specific phospho-sites is protein phosphatase 2 A (PP2A). Here we show that resveratrol, a polyphenol, significantly induces PP2A activity and reduces Tau phosphorylation at PP2A-dependent epitopes. The increase in PP2A activity is caused by decreased expression of the MID1 ubiquitin ligase that mediates ubiquitin-specific modification and degradation of the catalytic subunit of PP2A when bound to microtubules. Interestingly, we further show that MID1 expression is elevated in AD tissue. Our data suggest a key role of MID1 in the pathology of AD and related tauopathies. Together with previous studies showing that resveratrol reduces β-amyloid toxicity they also give evidence of a promising role for resveratrol in the prophylaxis and therapy of AD.

## Introduction

Alzheimer’s disease (AD) is the most common form of dementia and the most prominent neurodegenerative disorder associated with aging. One of the pathological hallmarks of AD is the development of paired helical filaments (PHFs) in the patients’ brains. PHFs have also been observed in AD-related tauopathies. Basis of PHFs is hyperphosphorylated Tau protein that, in a normo-phosphorylated status, associates with and stabilizes microtubules. Upon hyper-phosphorylation, Tau dissociates from the microtubules, sequesters normal Tau and other microtubule-associated proteins and thereby depolymerizes microtubules^1,2^.

Tau is differentially phosphorylated at over 30 sites in AD brains compared to normal. While several kinases including CDK5 and GSK3β are responsible for the phosphorylation of Tau, protein phosphatase 2A (PP2A) is the major phosphatase of Tau in the brain^3^. Interestingly, reduction of both expression and activity of PP2A has been described in brains of AD patients repeatedly^4–8^. This makes PP2A activity an interesting target for the development of a therapy for AD and related tauopathies.

A promising starting point for the development of PP2A-effective substances in the therapy of AD is its degradation process. We have shown previously that the catalytic subunit of PP2A (PP2Ac) and its regulatory α4 subunit interact with the microtubule-associated ubiquitin ligase MID1. After complex formation MID1 mediates the ubiquitin-specific modification of PP2Ac and its degradation by the proteasome, thereby providing a highly specific microtubule-centred regulation mode for PP2A^9^. Substances interfering with this interaction are interesting candidates for mediating an increase in microtubule-specific PP2A activity.

Resveratrol is a polyphenol that can be extracted from different plants including grapes and peanuts and is found particularly in red wine. It is sold as food additive in pharmacies and drug stores and has a very broad biological activity. Cardioprotective, anti-cancerogenic, as well as anti-inflammatory and beneficial metabolic effects have been described^10–12^. Furthermore, resveratrol is more and more being established as a neuroprotective drug after ischemic brain injury and in neurodegenerative disorders including Parkinson’s Disease^13,14^, AD^15,16^ and Huntington’s Disease^17,18^.

Mechanisms of action of resveratrol are numerous and largely unknown. However, it has been shown that resveratrol has anti-oxidant activity^19,20^, inhibits cycloxygenase activity^21,22^, ribonucleotide reductase^23^, protein kinase C^24^, DNA polymerase^ 25^ and has antiestrogenic properties^26,27^ and anti-platelet activity. Furthermore, it activates Sirt1, an NAD^+^-dependent protein deacetylase^28,29^ and also has been demonstrated to activate AMP kinase (AMPK)^30,31^, an important glucose sensor that inhibits acetyl-CoA carboxylase, thereby increasing oxidation of fatty acids and decreasing their synthesis.

Here we show that resveratrol treatment directly interferes with the MID1-α4-PP2A degradation complex by reducing MID1 protein expression *in vitro* and *in vivo*. This leads to an increase of microtubule-associated PP2A activity and a time- and dose-dependent dephosphorylation of Tau. Our data provide evidence for a promising effect of the polyphenol resveratrol in AD and related tauopathies.

## Results

### Resveratrol interferes with the MID1-α4-PP2A complex and reduces MID1 protein expression

**Figure 1:**
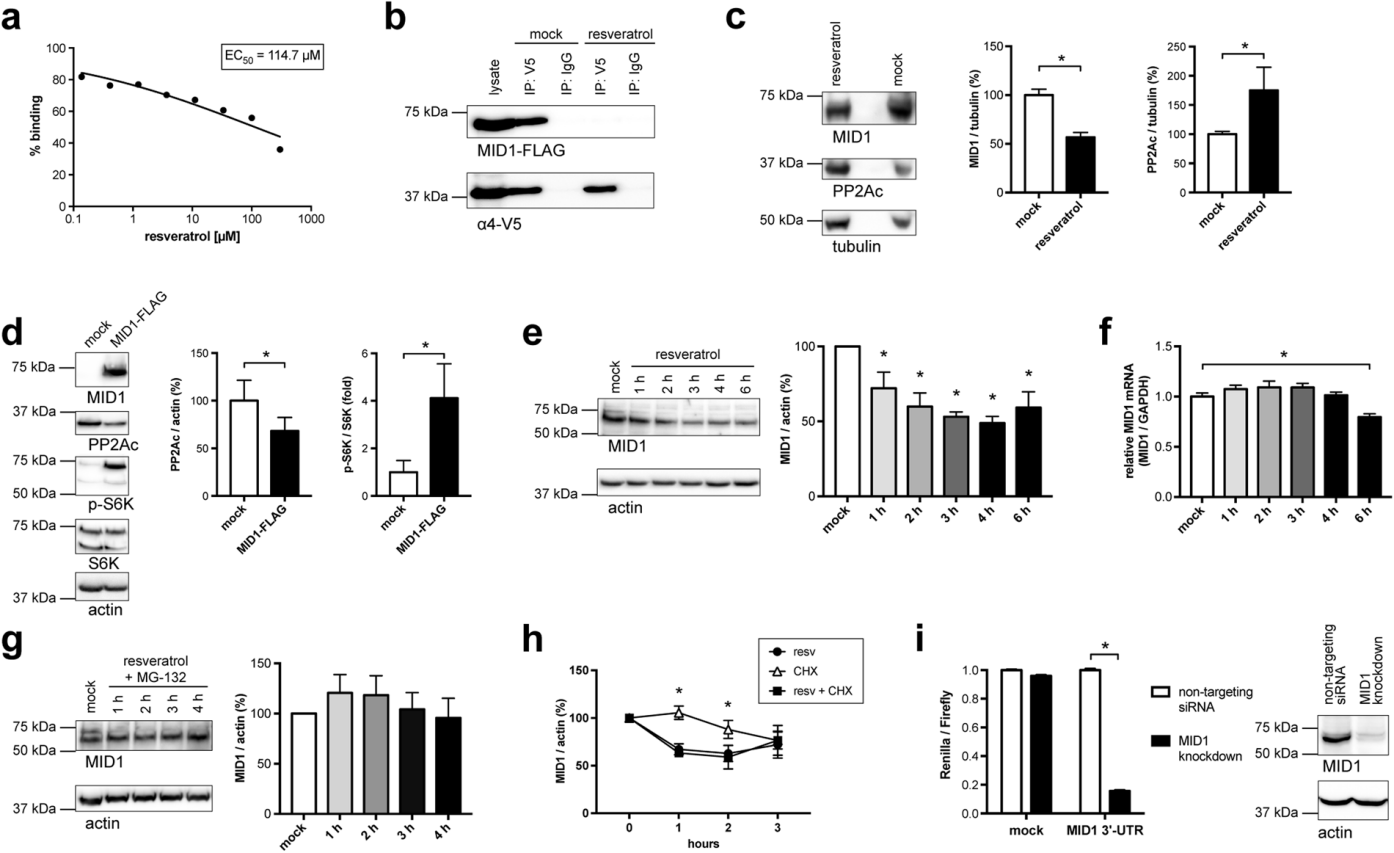
Resveratrol interferes with the MID1 complex assembly and reduces the MID1 transcript and protein level. (**a**) AlphaScreen protein-protein interaction assay. Resveratrol in various concentrations (starting at 300 µM) was incubated with MID1 (BBox1/2) and α4 (full-length) coupled to acceptor and donor beads respectively. Upon binding between MID1 and α4 the donor and acceptor beads come into proximity, resulting in a fluorescent signal that was quantified. (**b**) Co-immunoprecipitation of FLAG-MID1 and α4-V5 using V5 antibodies. Immunoprecipitates were incubated with or without resveratrol and subsequently washed. Immunoprecipitates were analysed on a western blot using FLAG- and V5- antibodies. (**c**) HEK293-β2a cells were treated with 100 µM resveratrol for 20 hours and analysed on western blots detecting MID1, PP2Ac and tubulin as loading control (n = 3). (**d**) HEK293T cells were transfected with FLAG-MID1 and analysed on western blots detecting MID1, PP2Ac, phospho-S6K, S6K, and actin as loading control. Right: quantification of western blots. Columns represent mean values +/− SEM (*p < 0.05) (n = 3). (**e**) HEK293T cells were treated with or without 100 µM resveratrol for 0–6 hours. Left: Cell lysates were analysed on western blots detecting MID1 and actin as loading control. Right: quantification of western blots. Columns represent mean values +/− SEM (*p < 0.05) (n = 3). (**f**) HEK293T cells were treated with or without 100 µM resveratrol for 0–6 hours. Expression levels of MID1 and GAPDH were analysed by real-time PCR. Samples were measured in triplicates and the relative MID1 expression normalized to GAPDH is shown. Graph represents mean values +/− SEM, (n = 3). (**g**) HEK293T cells were co-treated with 100 µM resveratrol and 10 µM MG132 for 0–4 hours. Left: Cell lysates were analysed on western blots detecting MID1 and actin as loading control. Right: quantification of western blots. Columns represent mean values +/− SEM (*p < 0.05) (n = 3). (**h**) HEK293T cells were treated with either the translation inhibitor cycloheximide (50 µg/ml), 100 µM resveratrol, or both substances in combination for increasing time intervals. The MID1 protein levels were analysed on western blots. The graph shows relative MID1 protein levels (normalized to actin), (*p < 0.05). (**i**) HEK293T cells were co-transfected with either non-silencing control or MID1 specific siRNAs directed against the coding region of MID1 in combination with a plasmid containing the MID1 3′-UTR downstream of the stop codon of renilla luciferase as well as firefly luciferase expressed from a different promoter. Relative light units of renilla normalized to firefly luciferase are shown. Columns represent mean values +/− SEM (*p < 0.01).

As PP2Ac constitutes the catalytic subunit of the most important Tau-phosphatase, inhibition of its degradation trigger, namely the MID1-α4 complex, offers promising possibilities to find novel treatment options for AD. In an AlphaScreen protein-protein interaction assay with the aim to identify disruptors of the MID1-α4 interaction we noted that the polyphenol resveratrol disrupts this interaction *in vitro* with an IC_50_ in the submillimolar range (Fig. 1a). To further validate that resveratrol has an influence on the MID1-α4 interaction we co-expressed FLAG-tagged MID1 and V5-tagged α4 in HEK293T cells. Cell lysates were then used for co-immunoprecipitation using V5-specific antibodies. Immunoprecipitates were incubated with or without resveratrol, washed, and analysed by western blot. In line with the data from the AlphaScreen co-precipitation of MID1 and α4 was clearly reduced after *in vitro* resveratrol treatment (Fig. 1b).

We then treated different cell lines with 100 µM resveratrol for 20 hours and tested for alterations in the MID1 complex and concomitant increases in PP2Ac. In several cell lines only weak effects could be observed, probably because potential changes in the MID1-affected microtubules-associated fraction of PP2Ac were obscured by the bulk cellular PP2Ac levels (data not shown). However, markedly increased levels of PP2Ac could be observed in a HEK293 derivative cell line (HEK293-β2a) when treated with resveratrol (Fig. 1c). This was accompanied by significantly reduced expression levels of the MID1 protein. In line overexpression of MID1 reduced PP2Ac levels significantly and also affected phosphorylation of the PP2A-target protein S6K (Fig. 1d).

Two possible scenarios could result in a reduction of MID1 protein expression after resveratrol treatment: (I) resveratrol could affect MID1 protein stability, or (II) resveratrol could affect the MID1 mRNA. To investigate the mechanism underlying the reduction of MID1 on both mRNA and protein level, we performed a time-course experiment with resveratrol in a time frame of 0 to 6 hours and quantified both the MID1 protein expression on western blots and the MID1 mRNA expression by quantitative real-time PCR. While after treatment with resveratrol MID1 protein and mRNA levels decreased, reduction rate of the MID1 protein was significantly faster than the reduction rate of the MID1 mRNA after resveratrol treatment (Fig. 1e,f). These data suggest that resveratrol affects the MID1 protein first, while the MID1 mRNA gets reduced as a secondary event. To test if resveratrol affects MID1 protein stability in a proteasome-dependent manner, HEK293T cells were co-treated with resveratrol and the proteasome inhibitor MG132 in a time-course experiment. Strikingly, inhibition of the proteasome blocked the reduction of MID1 after resveratrol treatment, indicating that disassembly of the MID1-α4 complex by resveratrol induces proteasomal degradation of MID1 (Fig. 1g). In line with an increased degradation of MID1 after resveratrol treatment, treatment with the translation inhibitor cycloheximide did not affect MID1 protein level after 1–2 hours of treatment nor did it further decrease MID1 levels upon double-treatment with resveratrol and cycloheximide (Fig. 1h).

The next question was how resveratrol, if it first affects MID1 protein level, leads to the secondary reduction of the MID1 mRNA after longer incubation times. As we had shown previously MID1 is an RNA binding protein binding its own mRNA through its 3′-UTR^32^. We therefore hypothesized that the MID1 protein has a stabilizing effect on its own mRNA through binding to its 3′-UTR. To show this the MID1 3′-UTR from base 2406–3697 (NM_000381.3) was cloned downstream of the stop codon of renilla luciferase. For normalization, firefly luciferase was expressed on the same plasmid from a different promoter. This construct was co-transfected with either non-silencing control or MID1 specific siRNAs directed against the coding region of MID1. These siRNAs target endogenous MID1 without affecting the renilla-MID1-3′-UTR construct. Upon depletion of endogenous MID1, a significant reduction of the expression of the MID1-3′-UTR-luciferase construct was observed (Fig. 1i), while the control construct without the MID1-3′-UTR was not affected by MID1 knockdown. These data suggest that MID1 indeed stabilizes its own mRNA by interacting with its 3′-UTR.

**Figure 2:**
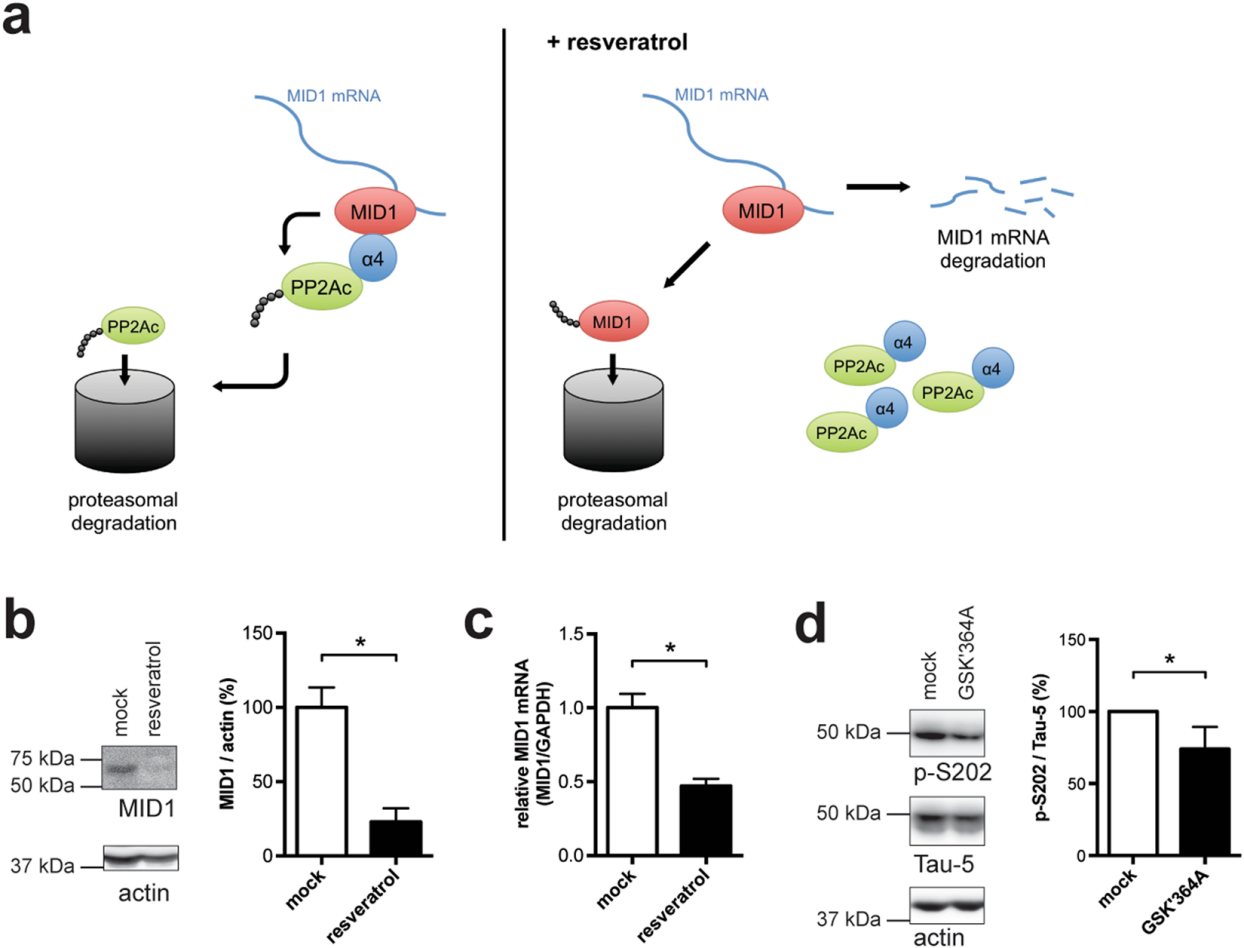
Resveratrol reduces the MID1 transcript and protein level in neurons. (**a**) Schematic showing the effect of resveratrol on MID1. Left: MID1 is a ubiquitin ligase that catalyses the ubiquitination of the catalytic subunit of PP2A (PP2Ac) and thereby stimulates proteasomal degradation of microtubule-associated PP2Ac. MID1 binds to and stabilizes its own mRNA. Right: Resveratrol treatment induces the proteasomal degradation of MID1, which stabilizes and activates PP2A at the microtubules. (**b**) Primary cortical neurons from wild-type mice were treated with 100 µM resveratrol for 20 hours. Cell lysates were analysed on western blots using antibodies detecting MID1 and actin as loading control (n = 3). (**c**) Primary cortical neurons from wild-type mice were treated with 100 µM resveratrol for 20 hours and expression levels of MID1 and GAPDH were analysed by real-time PCR. Samples were measured in quadruplicates and the relative MID1 mRNA expression normalized to GAPDH is shown. Columns represent mean values +/− SEM, (n = 4, *p < 0.002). (**d**) Primary cortical neurons from wild-type mice were treated with a peptide mimicking the MID1-α4 binding site (GSK′364A) or DMSO as negative control (mock) for 6 hours. Cell lysates were analysed on western blots using antibodies detecting Tau phosphorylation at S202, total Tau (Tau-5), and actin. Representative western blots and quantifications of several independent experiments are shown. Band intensities of phospho-Tau were normalized to total Tau (Tau-5). For each experiment the respective control sample was set to 100%. n = 11, *p < 0.001.

In summary, these data suggest a mechanism in which resveratrol stimulates PP2A activity by targeting the MID1 protein towards degradation via the proteasome. MID1 stabilizes its own mRNA. Dissociation of the MID1-PP2Ac complex leads to the proteasomal degradation of the MID1 protein followed by destabilization of its mRNA. Thereby, reduced expression of the ubiquitin ligase MID1 results in the stabilization of microtubule-associated PP2Ac (Fig. 2a).

### Resveratrol increases PP2A activity and dephosphorylates Tau

Tau is phosphorylated at multiple serine/threonine sites, many of which are PP2A-sensitive. To test if resveratrol also reduces the expression of MID1 in neuronal cells, murine primary cortical neurons were treated with resveratrol for 20 hours and MID1 protein expression was studied on western blots. A clear reduction of MID1 protein expression was observed after resveratrol treatment (Fig. 2b). Quantitative real-time PCR analyses from similarly treated cells revealed that MID1 mRNA levels were also significantly reduced after resveratrol treatment for 20 hours (Fig. 2c). The observed reduction of MID1 expression will activate PP2A towards its target proteins including Tau. To confirm that deregulation of the MID1 complex leads to dephosphorylation of Tau, primary cortical neurons were treated with a peptide that mimics the MID1-α4 binding site and thus will outcompete the binding of MID1 to PP2A. As expected, inhibition of MID1-PP2A complex assembly leads to significant decrease of Tau phophorylation (Fig. 2d).

**Figure 3:**
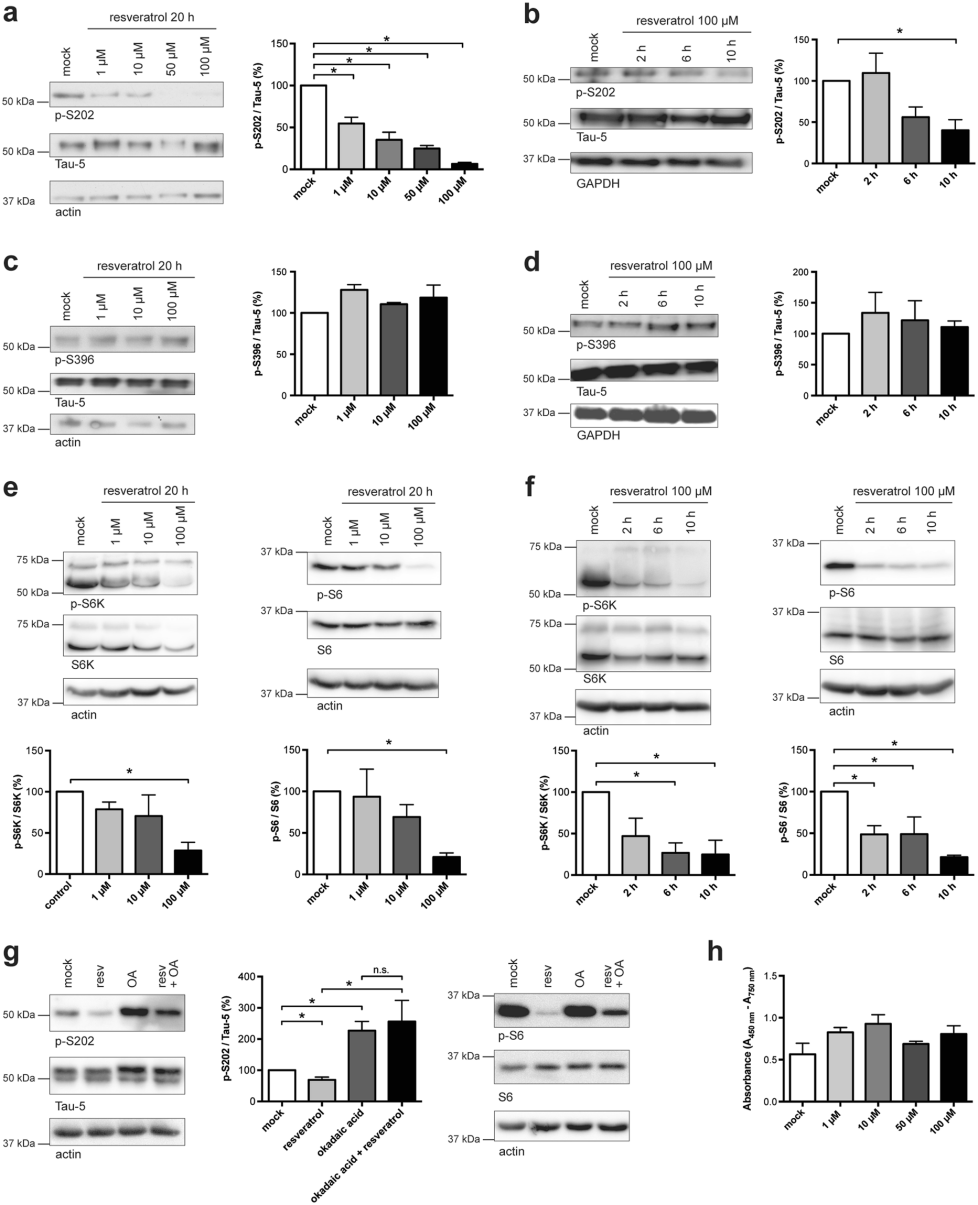
Resveratrol increases PP2A activity and dephosphorylates Tau at PP2A-sensitive sites in primary cortical neurons. Representative western blots and quantifications of several independent experiments are shown in a–g. Band intensities of phospho-Tau were normalized to total Tau (Tau-5). For each experiment the respective control sample was set to 100%. (**a**) Primary cortical neurons of wild-type mice were treated with increasing concentrations of resveratrol for 20 hours. Cell lysates were analysed on western blots using antibodies detecting Tau phosphorylation at S202, total Tau (Tau-5), and actin. n = 3, *p < 0.0001 (**b**) Primary cortical neurons from wild-type mice were treated with 100 µM resveratrol over increasing time intervals. Cell lysates were analysed on western blots using antibodies detecting Tau phosphorylation at S202, total Tau (Tau-5), and GAPDH. n = 6, *p < 0.05. (**c**) Primary cortical neurons of wild-type mice were treated with increasing concentrations of resveratrol for 20 hours. Cell lysates were analysed on western blots using antibodies detecting Tau phosphorylation at S396, total Tau (Tau-5), and actin or GAPDH as loading controls. n = 3. (**d**) Primary cortical neurons of wild-type mice were treated with 100 µM resveratrol over increasing time intervals (lower panel). Cell lysates were analysed on western blots using antibodies detecting Tau phosphorylation at S396, total Tau (Tau-5), and actin or GAPDH as loading controls. n = 3. (**e**) Primary cortical neurons from wild-type mice were treated with increasing concentrations of resveratrol for 20 hours. Cell lysates were analysed on western blots using antibodies detecting phospho-S6K (n = 3, *p < 0.05) and phospho-S6 (n = 5, *p < 0.05), total S6K as well as total S6, and actin. Band intensities of phospho S6/S6K were normalized to total S6/S6K. For each experiment the respective control sample was set to 100%. (**f**) Primary cortical neurons from wild-type mice were treated with 100 µM resveratrol over increasing time intervals. Cell lysates were analysed on western blots using antibodies detecting the PP2A targets p-S6K (n = 4, *p < 0.05) and p-S6 (n = 3, *p < 0.05), total S6K as well as total S6, and actin. (**g**) Primary cortical neurons from wild-type mice were treated with 100 µM resveratrol and/or the PP2A inhibitor okadaic acid (10 nM). Cell lysates were analysed on western blots using antibodies detecting Tau phosphorylation at S202, total Tau (Tau-5), phospho-S6, total S6 and actin (n = 4, *p < 0.05). (**h**) Cell viability is not affected by resveratrol. Primary neurons were treated with increasing concentrations of resveratrol for 20 hours and cell viability was measured in a WST-1 assay. Columns represent mean values +/− SEM (n = 6).

Next, we tested if the resveratrol-dependent reduction of MID1 also affects Tau phosphorylation. Primary cortical neurons from wild-type mice were treated with increasing concentrations of resveratrol for 20 hours. Cells were lysed and the phosphorylation pattern of Tau at selected PP2A-sensitive sites^2,33–35^ was analysed on western blots. Phosphorylation of the PP2A-sensitive Tau epitope p-S202 was significantly reduced in resveratrol treated cells in a concentration-dependent manner (Fig. 3a).

In a second series of experiments primary neurons from wild-type mice were incubated with 100 µM resveratrol over increasing periods of time. Cells were lysed and analysed for phosphorylation at the PP2A-sensitive epitope p-S202. A significant decrease of S202 phosphorylation was detected after 10 hours but not after 2 hours of incubation (Fig. 3c). Phosphorylation at S396, which is not an efficient PP2A target site^34^, however, remained unaffected by resveratrol treatment (Fig. 3c,d), clearly suggesting a PP2A-dependent mechanism of resveratrol activity. The influence of resveratrol on PP2A activity was analysed by monitoring the phosphorylation pattern of two direct targets of PP2A, p70-S6 kinase 1 (S6K) and the ribosomal protein S6. Phosphorylation of S6K and S6 was decreased in primary neurons in a time- and concentration-dependent manner after incubation with resveratrol (Fig. 3e,f).

To prove that the resveratrol-induced dephosphorylation of Tau is indeed PP2A-dependent, primary neurons were either treated with a PP2A inhibitor (okadaic acid) or with resveratrol or with both substances simultaneously. As expected, the resveratrol effect was blocked in the double treated cells, indicating that resveratrol influences Tau phosphorylation in a PP2A-dependent manner. Similarly, a partial block of the resveratrol effect by okadaic acid was seen on another PP2A target protein S6 (Fig. 3g).

A cell toxicity assay was used to prove that the observed effects were not caused by an increase in cell death after resveratrol treatment for 20 hours. Up to a concentration of 100 µM resveratrol had no detectable influence on cell viability (Fig. 3h). These observations were also confirmed in OLNt40 cells that stably express the longest isoform of human Tau (Supplementary Fig. 1).

### Resveratrol dephosphorylates tau *in vivo*

**Figure 4:**
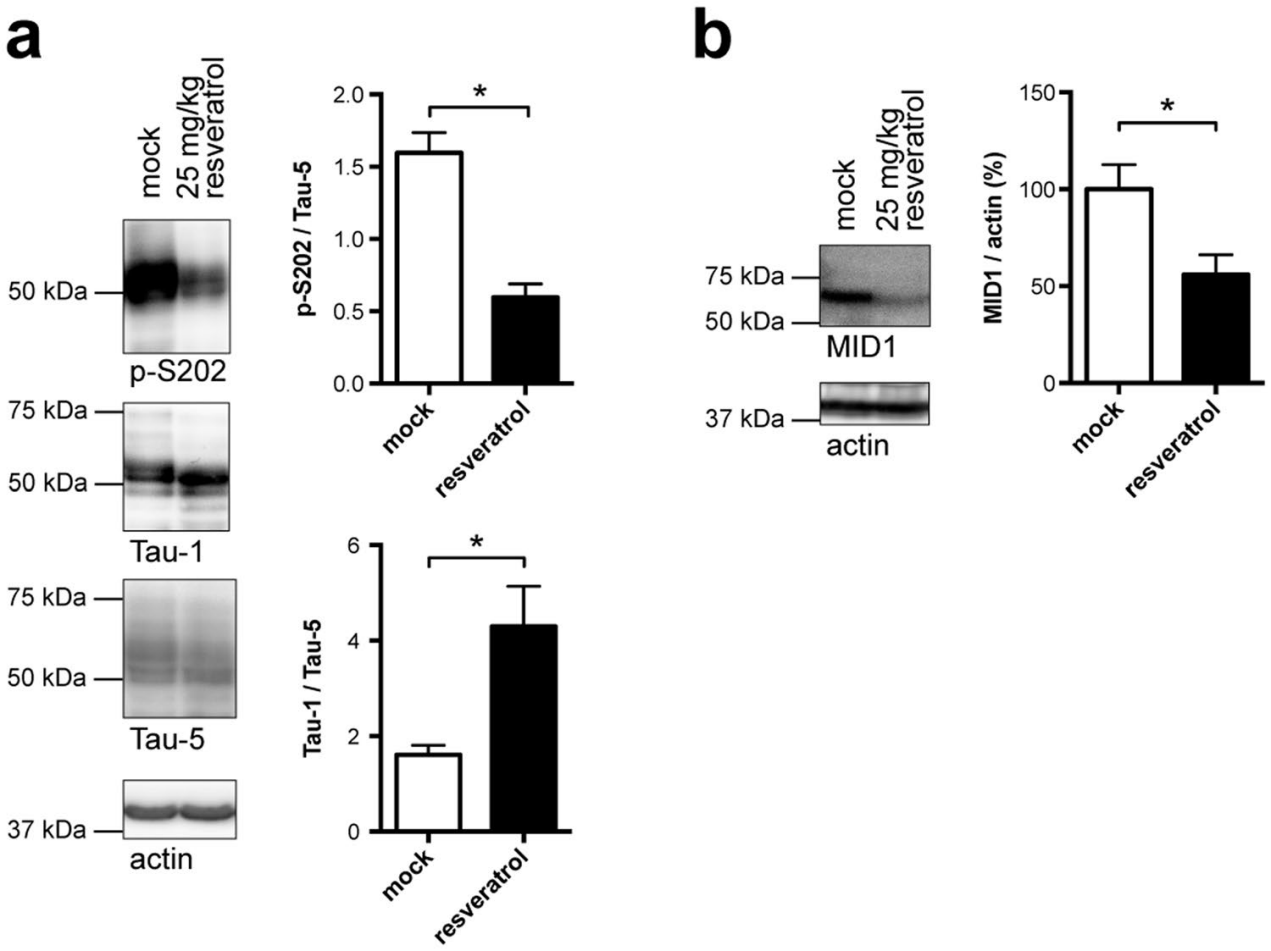
Resveratrol dephosphorylates Tau *in vivo*. Wild-type mice were treated for 2 weeks with 25 mg/kg resveratrol by daily intraperitoneal injections. (**a**) Brain lysates of these mice were analyzed on western blots using antibodies detecting phosphorylated Tau (p-S202), dephosphorylated Tau (Tau-1), total Tau (Tau-5), and actin. Columns represent mean values +/− SEM, (n = 4, *p < 0.05). (**b**) Brain lysates of mice described in (**a**) were analysed on western blots detecting MID1 and actin. n = 4.

To test if resveratrol is capable of reducing Tau phosphorylation *in vivo*, wild type mice were treated with resveratrol for 2 weeks by daily intraperitoneal injections (25 mg/kg). Brain lysates of these mice were analysed for Tau phosphorylation on western blots. As expected, multiple bands, corresponding to the different Tau isoforms expressed in adult brain were detected. Blots were analysed with an antibody detecting phosphorylated tau (p-S202) and an antibody detecting dephosphorylation at the S202 site (Tau-1). Quantification revealed that, similar to the cell culture models, a significant reduction of Tau phosphorylation at epitope S202 was observed in resveratrol treated mice (Fig. 4a). Intriguingly and supporting a substantial role of the MID1 ubiquitin ligase, this Tau dephosphorylation was accompanied by a significant reduction of MID1 protein levels in resveratrol treated mice (Fig. 4b).

### MID1 is overexpressed in patients with Aβ plaques and hyperphosphorylated Tau

**Figure 5:**
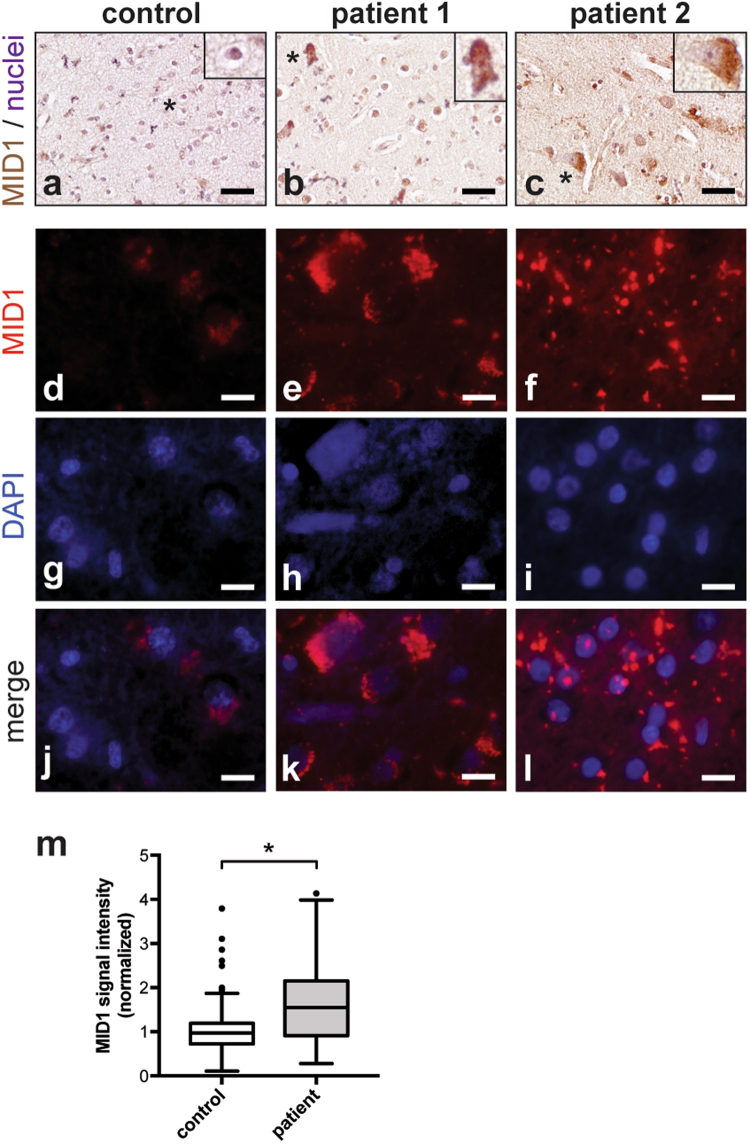
MID1 immunostaining of the temporal cortex from human control and patients with hyperphosphorylated Tau and Aβ plaque deposition. (**a–c**) MID1 immunohistochemistry. MID1 is shown in brown and the nuclei are denoted with eosin (blue). (**a**) Very little MID1 signal is observed in the control (no Alzheimer’s pathology or related clinical signs at the age of 79 years). (**b**) MID1 immunostaining is clearly present in patient 1, who was diagnosed clinically with AD and showed pathology of hyperphosphorylated Tau and intracellular Aβ plaque deposition (age 65 years). (**c**) Substantial MID1 signal is observed in patient 2, who had no clinical signs of AD at the age of 61 years, but showed significant pathology of Aβ plaques and neurofibrillary tangles. Scale bar = 200 µm. *Denote cells that were enlarged in the inset of each panel. (**d–l**) MID1 immunofluorescence staining. MID1 is stained in red, nuclei are visualized with DAPI (blue). (**d,g,j**) Very little MID1 signal is observed in the control. (**e**,**h**,**k**) MID1 immunostaining is clearly present in patient 1. (**f,i,l**) Substantial MID1 signal is observed in patient 2. Scale bar = 25 µm. (**m**) Quantification of MID1 signal intensity of samples shown in (**d–l**).

Our data suggest that MID1 plays a significant role in regulating PP2A activity and the phosphorylation of Tau in neurons. It therefore may be a key factor in the pathology of AD and other tauopathies. In brains of AD patients, both reduced PP2A activity and reduced PP2A expression had been shown previously^4–8^. To test the hypothesis that this reduction of PP2A activity may be at least in parts caused by MID1 hyperactivity, we performed immunohistochemistry staining of MID1 in post-mortem brain tissue of two patients with hyperphosphorylated Tau and Aβ plaques. Interestingly, while very little MID1 staining was observed in a healthy control sample, in both patients a clearly enriched MID1 staining was visible (Fig. 5). This increase in MID1 expression in AD strengthens the hypothesis that the MID1 protein complex is a promising drug target for AD therapy.

## Discussion

**Figure 6:**
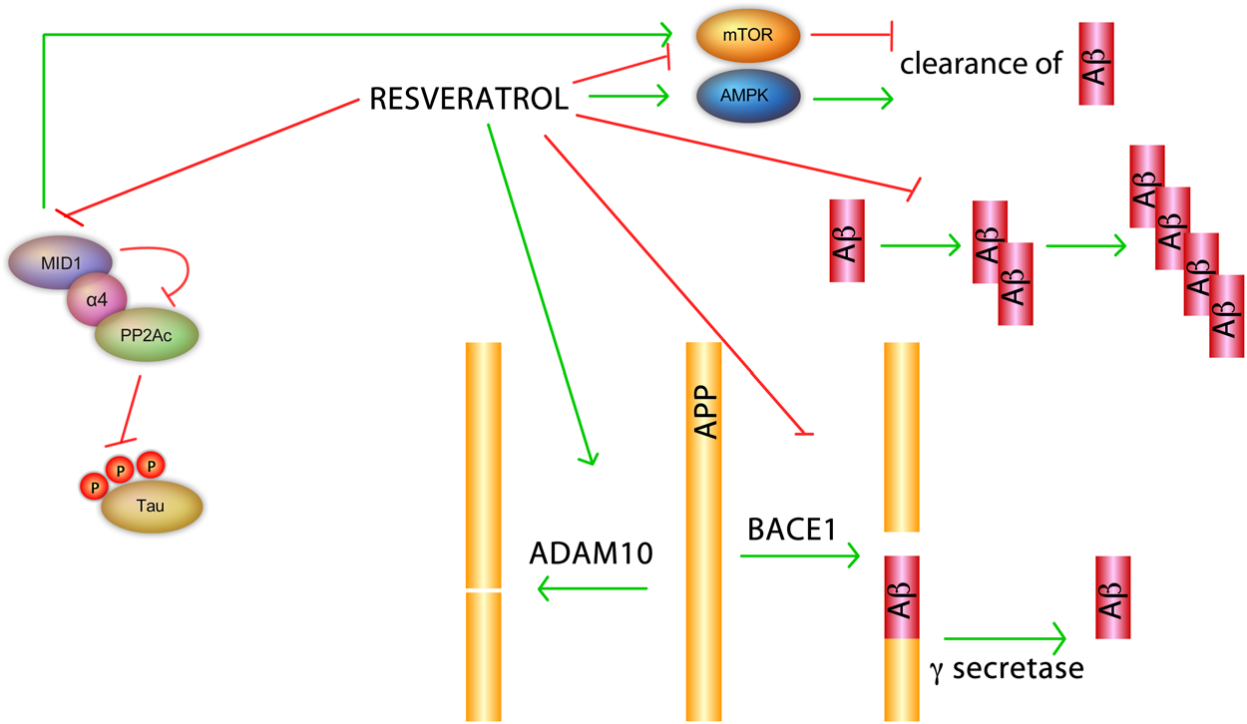
Resveratrol has multiple biological functions that are relevant for AD. Resveratrol acts on the neuropathological hallmarks of AD via multiple routes. Resveratrol inhibits the expression of MID1, thereby activating PP2A and dephosphorylating Tau. Additionally, MID1 induces the PP2A opposing kinase mTOR. Resveratrol induces degradation pathways by inhibiting mTOR signalling and inducing AMPK, thereby stimulating the clearance of Aβ. Resveratrol inhibits BACE1, resulting in decreased Aβ production. Resveratrol induces ADAM10, resulting in a preferential cleavage of APP via the non-amyloidogenic pathway.

One of the two major pathological hallmarks of AD is the formation of paired helical filaments (PHFs), protein aggregates formed by hyperphosphorylated Tau protein that dissociates from the microtubules. PP2A is the most important phosphatase that dephosphorylates Tau and thereby can prevent its microtubule-dissociation and the formation of PHFs. Activation of PP2A is a promising tool in the prevention and therapy of AD and related tauopathies. We here show that resveratrol destabilizes the microtubule-associated ubiquitin ligase MID1 *in vitro* and *in vivo*. Degradation of the MID1 protein destabilizes the MID1 mRNA resulting in even lower MID1 protein levels. MID1 plays a key role in the proteasomal degradation of PP2A^9^, its loss of function results in an accumulation of microtubule-associated PP2A and an increase of PP2A activity at the microtubules. Our data demonstrate that through proteasomal degradation of MID1 protein and the subsequent destabilization of its mRNA, resveratrol reduces MID1 expression, which is followed by a significant increase of microtubule-associated PP2A activity (shown by a decrease of phosphorylation of the PP2A targets S6K and S6). PP2A leads to the dephosphorylation of the microtubule-associated Tau protein at PP2A specific sites (Fig. 6). Therefore, our data support a beneficial role of resveratrol in AD pathology.

Resveratrol has diverse biological activities and it has been shown to play a significant neuroprotective role in several diseases like Parkinson’s disease^13,14^, Huntington’s disease^18^, amyotrophic lateral sclerosis^36^ and ischemic brain injury^37^. Also, with respect to AD, resveratrol has multiple beneficial effects. The underlying neuroprotective pathways are diverse. Most of them seem to interfere with senile plaques, which are composed of amyloid-β (Aβ) peptides. Aβ is derived from sequential proteolytic cleavage of the amyloid precursor protein (APP) by the β-secretase BACE1 and the α-secretase^38^. Resveratrol has been suggested to induce the α-secretase ADAM10, which outcompetes BACE1 and thereby reduces Aβ-production^15^. In addition resveratrol has been found to directly reduce BACE1 activity^39,40^. Resveratrol also induces protein degradation pathways for example it stimulates AMPK signalling and induces mTOR-dependent autophagy^41–45^. Furthermore, resveratrol can also directly act on Aβ aggregates, where it modulates Aβ confomers such that non-toxic high-molecular weight species are built^46^. Interestingly, the resveratrol-mediated reduction of Aβ increases life span and improves learning and memory^15,40^, reduces neuroinflammation^47^ and reduces oxidative stress^48^.

Possible influences of resveratrol on hyperphosphorylated Tau are far less studied. We show here that resveratrol efficiently induces dephosphorylation of the microtubule-associated protein Tau *in vitro* and *in vivo*. Our data are supported by observations that treatment with a polyphenolic derivative of resveratrol (pterostilbene) reduces Tau phosphorylation in mice and improves behaviour^49^. In the same study, however, the authors did not see an effect on Tau when using resveratrol. This is in contrast to our data and to the observations of Porquet *et al*., who also saw a decrease of phospho-Tau after resveratrol treatment in mice^15^. This can probably be explained by the usage of different mouse models and/or different treatment protocols (see also paragraph on bioavailability of resveratrol below).

An important question for the treatment of diseases of the nervous system is if or if not the polyphenol resveratrol passes the blood-brain barrier. This has been studied and demonstrated in laboratory animals^44,50^ and humans^51^. Of note, resveratrol not only passes but also protects the integrity of the blood-brain barrier in AD^47^. In a Class II clinical trial, resveratrol has been shown to be safe and well tolerated^51^. An adverse caveat of resveratrol in a therapeutic approach is its low bioavailability. Resveratrol is poorly soluble in water and is rapidly metabolized^52^. To avoid these problems Frozza *et al*. have used resveratrol incapsulated in loaded-lipid core nanoparticles. They could show that treatment with these nanoparticles significantly reduced neurotoxicity in rats that received intracerebroventricular injections of Aβ^53^. All these data together suggest that resveratrol is a promising lead compound for the prophylaxis and treatment of AD. Modified versions of resveratrol with higher bioavailability and increased target-efficacy will have to be developed in future studies.

In addition to the known modes of action of resveratrol, we show here that resveratrol destabilizes the MID1 ubiquitin ligase thereby significantly decreasing its protein levels in tissue culture and *in vivo*. MID1 is a negative regulator of PP2A that mediates the ubiquitin specific degradation of PP2A. Its loss of function leads to an increase of PP2A protein and activity levels^9^. At the same time MID1 stimulates the activity of the PP2A-opposing kinase mTOR^54^. Several bits of evidence have suggested an influence of resveratrol on PP2A and its opposing kinase mTOR before: in a large *in vitro* kinase screen S6K, a primary target of both PP2A and mTOR, was identified as a direct target of resveratrol^55^. In support of that we demonstrate here that resveratrol causes a significant reduction in phosphorylation of the two primary PP2A/mTOR targets S6K and S6 in primary neurons and Tau-expressing OLNt40 cells in a time and dose dependent manner. In the same cells the effects of resveratrol on S6K and S6 could be reversed by the PP2A inhibitor okadaic acid. This points towards a mechanism that directly involves (increased) PP2A activity.

Finally, both, reduced PP2A protein and activity levels have been described in brains of AD patients. We here demonstrate significantly elevated expression of MID1 in brains of AD patients compared to age-matched controls. According to previous and our present data that suggest a key role of the MID1 ubiquitin ligase in the processing of PP2A, one could speculate that high MID1 levels contribute to low PP2A levels and hyperphosphorylated Tau in AD.

Taken together our data suggest an important role for the microtubule-associated MID1 ubiquitin ligase in the regulation of neuronal PP2A activity and the dephosphorylation of Tau. Furthermore, we demonstrate a new mode of action of resveratrol that supports potentially beneficial effects of resveratrol in AD.

## Methods

### Primary cultures

Primary cortical neurons were isolated from wild-type mouse embryos at day E14.5. Cortices were dissociated by incubation with Trypsin/EDTA for 7–10 min at 37 °C. Cells were plated in neurobasal medium (Invitrogen) containing B27 supplement (Invitrogen) at a density of 8 × 10^5^ onto 0.2 mg/ml poly-D-lysin (Sigma) and 2 μg/ml Laminin (Sigma) coated 6-well plates. Neurons were incubated at 37 °C with 5% CO_2_. Thirty minutes after seeding a complete change of medium was performed. Cells were treated after 4 days in culture.

### Treatments

Cells were incubated with the respective substances as follows: resveratrol (Sigma) at final concentrations up to 100 µM for 1 to 24 hours, MG132 at a final concentration of 10 µM, the translation inhibitor cycloheximide at a final concentration of 50 µg/ml and the PP2A inhibitor okadaic acid at a final concentration of 10 nM. Either HEK293T or HEK293-β2a cells were used. HEK293-β2a is a HEK293 cell line that was transfected stably with β-2-andrenergic receptor. This cell line is a kind gift and was originated by Dr. Eduard Stefan (Institute of Biochemistry, University of Innsbruck).

Primary cortical neurons from wild-type mice were treated with 5 µM of a peptide mimicking the MID1-α4 binding site (GSK′364A) or DMSO as negative control (mock) for 6 hours. The peptide (GSK′364A) containing a 29-residue sequence from α4 (AQAKVFGAGYPSLPTMTVSDWYEQHRKYG) with an N-terminal sequence derived from HIV-TAT protein (RKKRRQRRR) was supplied by Cambridge Research Biochemicals (Billingham, UK). It was synthesised using standard automated solid-phase peptide synthesis via the Fmoc/tBu strategy. Cleavage from the resin was performed using 95% trifluoroacetic acid. Crudes were purified by preparative high-performance liquid chromatography (HPLC), freeze dried and characterised by high-performance liquid chromatography (HPLC) and matrix-assisted laser desorption ionization time-of-flight (MALDI-TOF) mass spectrometry.

Female wild type C57BL/6 mice at an age of 12 weeks were treated for 2 weeks with 25 mg/kg resveratrol by daily intraperitoneal injections. Resveratrol was dissolved in DMSO at a concentration of 25 mg/ml. Animals were sacrificed by cervical dislocation and brains were snap-frozen in liquid nitrogen and broken up using a mortar. All procedures were in compliance with german animal protection law and were approved by the competent authorities (Landesamt für Naturschutz und Verbraucherschutz Nordrhein-Westfalen; AZ 87-51.04.2011.A049/01).

### Western Blot

Cell pellets were homogenized in Magic-Mix (48% urea, 15 mM Tris-HCl pH 7.5, 8.7% glycerol, 1% SDS, 0.004% bromophenol blue, 143 mM 2-mercaptoethanol) or Buffer B (4% SDS, 25 mM EDTA, 2% 2-mercaptoethanol, 20% glycerol, 100 mM Tris pH 6.8), sonicated and boiled for 5 min at 95 °C. Proteins were resolved on 8 or 10% SDS gels and blotted onto PVDF membranes (Roche). The resulting bands were quantified using the Imagequant 5.2 software. Statistical analyses were performed using the GraphPad Prism software. Columns shown in graphs represent mean values +/− SEM. Data were analysed by multiple t-tests or one-way ANOVA with post-hoc Dunnett’s test to accommodate for multiple comparisons.

### Antibodies

Antibodies used in this study were purchased from the following companies: Tau-5 (Biosource), anti-human PHF p-S202 (Thermo scientific), Tau p-Ser356 (Biosource), Tau p-S262 (Biosource), Tau p-S396 (Sigma), actin (Sigma), phospho-S6 ribosomal protein p-Ser241/244 (Cell signalling), S6 ribosomal protein (Cell signalling), S6K (Cell signalling), p-S6K p-T421/p-S424 (Cell signalling), mTOR (Cell signalling), HRP-anti-rabbit (Amersham), HRP-anti-mouse (Dianova), FLAG-HRP (SIGMA), V5 (Invitrogen). Generation of anti-α4 was described previously^9^. For production of polyclonal MID1 antibodies MID1-peptides were synthesized (amino acids 84–113) and used for immunisation of rabbits (PINEDA). Eight weeks after immunisation high-titre sera were collected and affinity purified using the peptide coupled to SulfoLink Coupling Resin (Thermo Scientific) following the manufacturer’s instructions. The purified antibodies were then validated on western blots of cell lysates from cells that underwent MID1 siRNA mediated knockdown, as well as in western blot experiments in which peptide-blocking was performed (data not shown).

### WST-1 Assay

Cells were grown in a 96-well plate and treated with increasing concentrations of resveratrol for 20 hours. Cell viability was then measured using the WST-1 reagent (Roche) according to the manufacturer’s instructions. In brief, cells were incubated with the ready-to-use WST-1 reagent, which can be cleaved to a soluble formazan by cellular processes dependent on NAD(P)H. The formazan dye was quantified in an ELISA reader and this signal directly correlates to the number of metabolic active cells in the culture.

### OLN-t40 cells

OLN-t40 cells are a permanent oligodendroglia cell line derived from primary rat brain glial cultures, stably expressing the longest human Tau isoform, which has been established by Goldbaum *et al*.^56^. Cells were kept in DMEM supplemented with 10% heat-inactivated fetal calf serum, 2 mM glutamine, 50 U/ml penicillin, 50 µg/ml streptomycin, and 1 mg/ml G418. OLN-t40 were transfected with FLAG-MID1 using Lipofectamine 2000 (LifeTechnologies) according to the manufacturer’s instructions.

### Co-immunoprecipitation

For co-immunoprecipitation experiments, HEK293T cells were plated in 75 cm^2^ flasks at a density of 8 × 10^5^ one day prior transfection. Cells were transfected with FLAG-MID1 and α4-V5 using Polyfect (Qiagen) according to the manufacturer’s instructions. 48 hours after transfection cells were lysed using precellys in IP-buffer [containing 50 mM Tris pH 7.5, 2.5 mM MgCl_2_, 100 mM NaCl, 1 mM DTT, Complete protease inhibitor cocktail (Roche)]. Immunoprecipitation was carried out using V5-specific antibodies or unspecific mouse IgG as negative controls in combination with Protein A-Agarose (Roche) following the manufacturer’s instructions. Antibody-bound proteins were incubated with or without resveratrol (100 µM) for 2 hours and subsequently immunoprecipitates were washed with IP-buffer with or without resveratrol for 2 hours and immunoprecipitates were analysed on western blots.

### Real-time PCR

RNA was isolated using the RNeasy Mini Kit (Qiagen). cDNA synthesis was done with the TaqMan reverse transcription reagents kit (Applied Biosystems) and real-time PCR was carried out using the SYBRGreen PCR master mix (Applied Biosystems). Primer sequences see Table S1.

### MID1 knockdown and luciferase assays

7.5 × 10^4^ HEK293T cells (24-well plate) were transfected with Oligofectamine reagent (Invitrogen) and siRNA oligonucleotides (Table S1) according to the manufacturer’s instructions. 24 hours after knockdown cells were transfected with Lipofectamine 2000 (Invitrogen) and psiCHECK-2 luciferase reporter plasmids. 24 hours after psiCHECK transfection, cells were harvested in passive lysis buffer. Firefly and renilla luciferase activities were measured using the Dual-Luciferase Assay system (Promega) and a FLUOstar Omega luminescence microplate reader (BMG Labtech).

### Immunohistochemistry

Human brain samples were obtained from The National Disease Research Interchange (NDRI). NDRI serves as a Human Tissue and Organ for Research Resource (HTORR). Every researcher obtains NDRI approval prior to receiving human samples. NDRI receives funding and oversight from United States federal agencies, including the Office of the Director at the National Institutes of Health (NIH), to support the recovery and distribution of donated human organs and tissues for use in research programs across multiple disciplines. NDRI works with US-based organ procurement organizations (OPOs), tissue banks, eye banks, hospitals, and independent recovery personnel to recover project-driven biospecimens. In all cases, the donors or next-of-kin have provided informed consent to procure biospecimens for biomedical research. Research on human samples was performed following The Code of Ethics of the World Medical Association (Declaration of Helsinki). Samples were manipulated following the universal standards for working with human samples and as directed by the Institutional Review Board of The University of Texas Medical School at Houston (IRB approval # HSC-MS-14-0608).

Patient 1 showed clinical signs of AD and dementia was diagnosed 4 years before death at the age of 65 years. In this patient severe Aβ plaque the presence of hyperphosphorylated Tau was observed. Patient 2 showed extensive Aβ plaque accumulation and the presence of hyperphosphorylated Tau and neurofibrillary tangles but had no clinical signs of AD at the age of 61 years. The control sample had no AD pathology or related clinical signs (79 years).

Formalin-fixed samples from the temporal cortex were immunostained. Sections were deparaffinised and digested with pepsin, 1 mg/ml in 0.1 N HCl for 30 minutes at room temperature for antigen retrieval. To reduce non-specific staining sections were incubated with 10% Normal Donkey Serum for 1 hour. The sections were incubated overnight at 4 °C with anti-MID1 antibody (Abcam ab70770), followed by incubation with donkey-anti-rabbit biotin conjugated secondary antibody. Peroxidase reaction was visualized using DAB Kit (Vector) according to the manufacturer’s instructions. Nuclei were stained by eosin staining. Finally, sections were dehydrated in graded ethanol and mounted with Cytoseal 60.

For immunofluorescence stainings, sections were deparaffinised and digested with pepsin, 1 mg/ml in 0.1 N HCl for 15 minutes at room temperature for antigen retrieval. Sections were blocked with 10% Normal Donkey Serum for 1 hour and incubated overnight at 4 °C with anti-MID1 antibody (Abcam ab70770), followed by incubation with alexa fluor A2120-conjugated secondary antibody for 1 hour. After washing, sections were mounted with ProLong Gold Antifade Mountant with DAPI (ThermoFisher).

We used a generalized linear model to analyze differences in red channel intensities between patient and control groups. This statistical model accounts for confounding subject-dependent effects. Due to non-negative nature of intensities, the data is modeled using the gamma distribution.

### AlphaScreen protein protein interaction assay

The interaction between MID1 and α4 was studied in an AlphaScreen (Perkin Elmer) protein protein interaction assay using a GST-tagged MID1-construct consisting of the two BBox domains (which contains the binding site for α4) and biotinylated α4. The AlphaScreen kit (Perkin Elmer) that was used to measure the binding between MID1 and α4 contained streptavidin donor and Ni-chelate acceptor beads. In this assay, α4 was coupled to the donor beads, and MID1 was bound to the acceptor beads. Upon binding between MID1 and α4 the donor and acceptor beads come into proximity, and the excitation of the donor will result in generation of a fluorescent signal. The purified proteins (at a final concentration of 50 nM) were incubated with or without different doses of resveratrol for 2 hours and then the AlphaScreen beads (at a final concentration of 20 µg/ml) were added. The reaction was incubated over night at room temperature in a buffer containing 50 mM Phosphate pH 7.8, 150 mM NaCl, 1% DMSO, 0.01% Triton X-100, and 0.1% BSA.

## Electronic supplementary material

Supplementary information
