# Supplementary material for: Resveratrol induces dephosphorylation of Tau by interfering with the MID1-PP2A complex
Source: Sci Rep. 2017 Oct 23;7:13753. doi: 10.1038/s41598-017-12974-4 (PMC5653760; doi:10.1038/s41598-017-12974-4)
Supplement: Supplementary file 1 — Supplementary information [file 41598_2017_12974_MOESM1_ESM.pdf]

Title: Resveratrol induces dephosphorylation of Tau by interfering with the MID1-PP2A complex

Authors: Susann Schweiger[*,a,#], Frank Matthes[*,b], Karen Posey[c], Eva Kickstein[d], Stephanie Weber[b], Moritz M. Hettich[b], Sandra Pfurtscheller[e], Dan Ehninger[b], Rainer Schneider[e], Sybille Krauß[#,b]

Supplementary Figures

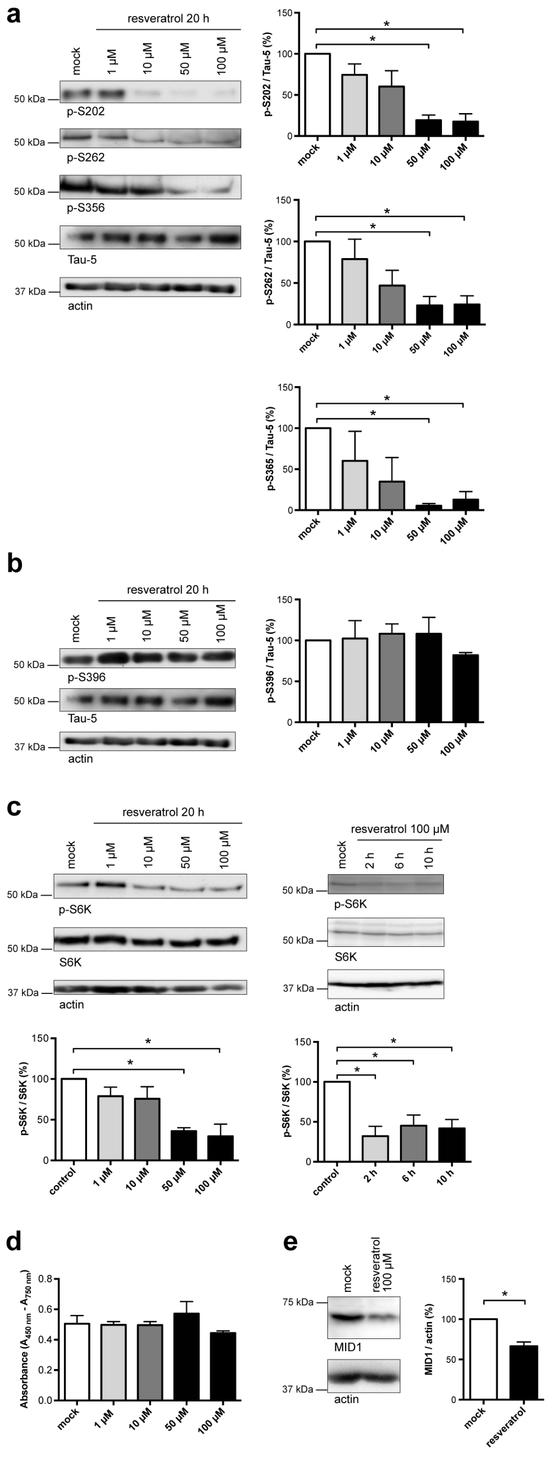

Supplementary Figure 1:

Resveratrol increases PP2A activity and dephosphorylates Tau at PP2A-sensitive sites in OLN-t40 cells.

(a) OLN-t40 cells were treated with increasing concentrations of resveratrol for 20 hours. Cell lysates were analysed on western blots using an antibody detecting Tau

phosphorylation at S202 (n = 4), S262 (n = 4), and S356 (n = 3), total Tau (Tau-5), and actin. * $p < 0.05$ (b) OLN-t40 cells were treated with increasing concentrations of resveratrol for 20 hours. Cell lysates were analysed on western blots using antibodies detecting Tau phosphorylation at S396, total Tau (Tau-5), and actin as loading controls. n = 3. (c) OLN-t40 cells were treated with increasing concentrations of resveratrol for 20 hours (n = 4, * $p < 0.05$) or with 100 µM resveratrol over increasing time intervals (n = 5, * $p < 0.05$). Cell lysates were analysed on western blots using antibodies detecting phospho-S6K, total S6K, and actin. (d) Cell viability is not affected by resveratrol. OLN-t40 cells were treated with increasing concentrations of resveratrol for 20 hours and cell viability was measured in a WST-1 assay. Columns represent mean values +/- SEM. (n = 9). (e) Resveratrol reduces MID1 protein level. OLN-t40 cells were treated with 100 µM resveratrol for 20 hours. Cell lysates were analysed on western blots using antibodies detecting MID1 and actin as loading control (n = 3, * $p < 0.05$).
